# Genetic and physical interaction of Meis2, Pax3 and Pax7 during dorsal midbrain development

**DOI:** 10.1186/1471-213X-12-10

**Published:** 2012-03-05

**Authors:** Zsuzsa Agoston, Naixin Li, Anja Haslinger, Andrea Wizenmann, Dorothea Schulte

**Affiliations:** 1 Institute of Neurology (Edinger Institute), J. W. Goethe University Medical School, Heinrich Hoffmannstr. 7, 50628 Frankfurt, Germany; 2 Max Planck Institute for Brain Research, Deutschordenstr. 46, 50628 Frankfurt, Germany; 3 JRG Developmental Neurobiology, Biocenter, Julius Maximilians University, Am Hubland, 97074 Würzburg, Germany; 4 Department of Pharmacology, Howard Hughes Medical Institute, and the Institute for Stem Cell and Regenerative Medicine, University of Washington School of Medicine, Seattle, WA98109, USA; 5 Department of Neurosurgery, Tianjin Medical University General Hospital, Tianjin 300052, China; 6 Helmholtz Center Munich, German Research Center for Environmental Health, Neuherberg, Germany; 7 Institute of Anatomy, Experimental Embryology, Eberhard Karls University, Tübingen, Germany

## Abstract

**Background:**

During early stages of brain development, secreted molecules, components of intracellular signaling pathways and transcriptional regulators act in positive and negative feed-back or feed-forward loops at the mid-hindbrain boundary. These genetic interactions are of central importance for the specification and subsequent development of the adjacent mid- and hindbrain. Much less, however, is known about the regulatory relationship and functional interaction of molecules that are expressed in the tectal anlage after tectal fate specification has taken place and tectal development has commenced.

**Results:**

Here, we provide experimental evidence for reciprocal regulation and subsequent cooperation of the paired-type transcription factors *Pax3, Pax7 *and the TALE-homeodomain protein *Meis2 *in the tectal anlage. Using in ovo electroporation of the mesencephalic vesicle of chick embryos we show that (i) *Pax3 *and *Pax7 *mutually regulate each other's expression in the mesencephalic vesicle, (ii) *Meis2 *acts downstream of *Pax3/7 *and requires balanced expression levels of both proteins, and (iii) Meis2 physically interacts with Pax3 and Pax7. These results extend our previous observation that *Meis2 *cooperates with *Otx2 *in tectal development to include Pax3 and Pax7 as Meis2 interacting proteins in the tectal anlage.

**Conclusion:**

The results described here suggest a model in which interdependent regulatory loops involving *Pax3 *and *Pax7 *in the dorsal mesencephalic vesicle modulate *Meis2 *expression. Physical interaction with Meis2 may then confer tectal specificity to a wide range of otherwise broadly expressed transcriptional regulators, including Otx2, Pax3 and Pax7.

## Background

Progressive regionalization events subdivide the early developing neural tube into a series of distinct units, which are marked by the expression of specific combinations of transcriptional regulators and signaling molecules. Expression of many of these proteins broadly overlaps at early embryonic stages, but progressively restricts later in embryogenesis due to a series of positive and negative regulatory events. This leads to the generation of molecularly defined territories, which subsequently differentiate into anatomically and functionally different brain structures. A well-studied example for such regionalization events is the development of the mesencephalic alar plate, the anlage of the optic tectum in non-mammalian vertebrates or of the superior colliculus in mammals. The optic tectum develops from the caudal most part of the dorsal aspect of *Otx2 *expression domain. *Otx2 *expression is an essential prerequisite for the development of all anterior brain structures, which is evident in the lack of fore- and midbrain derived structures in *Otx2 *mutant mice [1-3]. Tectal development is tightly linked to the activity of the mid-hindbrain boundary (MHB) organizer, a group of cells located at the junction between the mesencephalic and metencephalic vesicles. Cells of the MHB organizer secrete long-range and short-range signaling molecules, which are necessary and sufficient for the development of the adjacent mid- and hindbrain structures [4]. Transplantation of the MHB region into the diencephalon, mesencephalon or rhombencephalon elicits the ectopic expression of mid-/hindbrain markers and the formation of ectopic polarized mesencephalic and cerebellar structures surrounding the graft [5-8]. This activity can be mimicked by local application of *Fgf8*, a secreted protein normally expressed within the MHB organizer territory [9,10]. Induction and maintenance of *Fgf8 *expression and MHB organizer activity depends on multileveled genetic interactions of transcription factors and signaling molecules, which include (among others) the secreted molecules *Wnt1, Wnt3a *and *Wnt10b*, the nuclear proteins *Pax2/3/5/7/8, En1, En2*, and *Lmx1b*, all of which act in positive feedback loops with *Fgf8 *[4]. Conversely, feedback antagonists of *Fgf8 *signaling such as *Sef, Spry1, Spry2 *and *Mkp3 *confine the organizer activity to a narrow ring of cells at the mid-hindbrain junction [4]. Positive and negative autoregulation thus shapes and maintains the MHB organizer.

**Figure 1:**
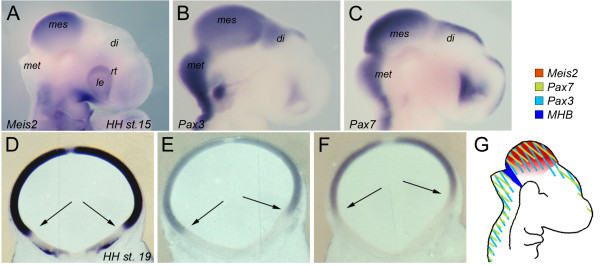
***Meis2, Pax3 *and *Pax7 *are expressed in nested domains in the HH15-19 chick midbrain**. (**A-G**) Expression of *Meis2 *(**A, D**), *Pax3 *(**B, E**) and *Pax7 *(**C, F**) as detected by in-situ hybridization on HH15 whole chick embryos (**A-C**) or neighboring vibratome cross sections through a HH19 mesencephalic vesicle (**D-F**). (**G**) Schematic summary of the expression patterns. di: diencephalic vesicle; le: lens; mes: mesencephalic vesicle; met: metencephalic vesicle; rt: retina. The arrows in (**D-F**) mark the ventral border of the respective expression domains. Panel (**D**) was taken from [17].

Two transcription factors reported to contribute to MHB organizer maintenance are the paired-box transcription factors *Pax3 *and *Pax7 *[11,12]. Both proteins share extensive homologies in protein sequence and expression patterns and are therefore believed to have arisen from a gene duplication event [13]. The importance of *Pax3 *in dorsal neural tube and neural crest patterning and differentiation is evident in the human syndromes associated with Pax3 mutations (Waardenburg syndromes type I and type III) as well as in mouse Splotch mutants. By contrast, Pax7 mutant mice do not display major defects in central nervous system development, which suggests a significant degree of functional overlap of the two Pax proteins [14,15]. In fact, knock-in of Pax7 can rescue the central nervous system and neural crest defects associated with the Pax3/Splotch mutant phenotype [16]. In chick embryos, *Pax3 *and *Pax7 *are expressed from the ten somite stage onwards in nested domains within the dorsal neural tube. By the 25-26 somite stage, mesencephalic *Pax3 *expression extends more ventrally than that of *Pax7*, whereas only *Pax7 *expression reaches rostrally into the telencephalic vesicle [[11]; and Figure 1). Although expression of both genes is not specific for the mid-hindbrain territory, ectopic expression of either one induced expression of MHB organizer associated genes including *Fgf8 *and *En2 *and elicited development of ectopic tectal structures [11].

We have previously reported a pivotal role for *Meis2 *in tectal development [17]. Meis2 belongs to the TALE (three amino acid loop extension) class of homeodomain containing proteins, which function as regulators of cell proliferation and differentiation of various tissues and organs during development. Meis family proteins form dimeric and trimeric complexes with other transcription factors, including the closely related Pbx family, members of the HOX clusters, several other homeodomain containing proteins and some basic helix-loop-helix (bHLH) proteins [18]. Mesencephalic *Meis2 *expression commences around the 19-20 somite stage and thus later than *Pax3 *and *Pax7*. Within the mesencephalic vesicle, *Meis2 *is largely confined to the tectal anlagen with sharp expression boundaries to the diencephalon and the MHB territory [17]. *Meis2 *is both necessary and sufficient for tectal development: introduction of a function blocking form of *Meis2 *into the mesencephalic vesicle abolishes normal tectal development, whereas a single, transient transfection of *Meis2 *in the diencephalic vesicle triggers the development of ectopic tectal structures. *Meis2 *lies downstream of *Fgf8 *in the hierarchy of tectum inducing genes, autoregulates its own expression and thereby stabilizes tectal fate [17,19]. The underlying mechanism, however, is only partially understood. One unique feature of *Meis2 *is its ability to induce a di- to mesencephalic fate change without any noticeable induction of *Fgf8 *expression. Instead, Meis2 directly interacts with Otx2 and competes with the Groucho co-repressor protein Tle4 for binding to Otx2, thereby releasing Otx2 from Tle4 mediated repression [17]. Whether Meis2 also associates with transcriptional regulators of tectal development other than Otx2 and what molecules regulate *Meis2 *expression levels within the mesencephalic alar plate is not known yet. This is important, since *Meis2 *autostimulates its own expression in the tectal anlage [17]. Some restraint needs therefore to be in place to prevent exuberant autoactivation of *Meis2*. So far, the nature of any inhibitory activity on *Meis2 *expression within the developing optic tectum remained elusive.

Here, we report a series of gain of function and biochemical experiments performed in chick embryos, which suggest that i) ectopic *Pax7 *expression induces *Pax3*, ii) elevated expression levels of *Pax3 *reduce expression of *Pax7 *and *Meis2*, but at different concentrations, which suggests *Meis2 *expression requires a specific concentration threshold of *Pax3 *and *Pax7*, and iii) both paired domain proteins form heteromeric complexes with Meis2. These results show that interdependent regulatory loops involving *Pax3, Pax7 *and *Meis2 *exist in the tectal anlage and argue for a more general role of *Meis2 *as co-factor of different transcriptional regulators of tectal development.

## Results

### *Meis2, Pax3 *and *Pax7 *are expressed in nested territories in the tectal anlage

A hallmark of early mid-/hindbrain fate specification is the mutual cross-regulation of proteins, which are expressed across the MHB organizer. Much less is known, however, about genetic interactions that may occur in the tectal anlage after initial fate specification has taken place. As a first step, we therefore compared the expression domains of *Pax3, Pax7 *and *Meis2*. Consistent with previous reports, we detected *Pax3 *and *Pax7 *transcripts at Hamburger Hamilton (HH) stage 15 (24-27 somites) in the alar plates of the spinal cord, and of the met-, mes- and diencephalic vesicles extending anteriorly to include prosomere 1 (Figure 1B, C, G) [11,20-24]. *Pax7 *was additionally expressed along the telencephalic midline. In contrast, *Meis2 *expression was absent from the met-, di- and telencephalon, but strongly expressed in the mesencephalic vesicle (Figure 1A, G) [17]. One day later, at HH19 (37-40 somites), *Pax3, Pax7 *and *Meis2 *transcripts were still abundant in the tectal anlage, as in-situ hybridization on adjacent vibratome sections showed (Figure 1D-F).

### Overexpression of *Pax7 *induces *Pax3 *but represses *Meis2- *and *ephrin-B1*

**Figure 2:**
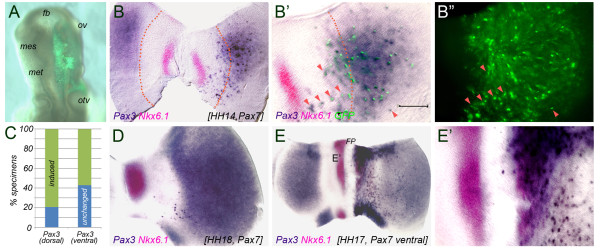
***Pax7 *induces *Pax3 *expression in the mesencephalic vesicle**. (**A**) Representative example of pMES-*Pax7 *electroporation into the mesencephalic vesicle. GFP expression, indicative of transgene expression, is restricted to the right half of the neural tube 4-6 hours later. (**B-B**") Flat mount preparation of a HH14 chick neural tube, 16 hours after electroporation of 2 μg/μl pMES-*Pax7 *into the mesencephalic vesicle; *Pax3 *expression is in dark blue, *Nkx6.1 *expression in pink, GFP in green. The dotted lines mark the dorso-ventral boundary, the arrow heads indicate representative examples of ectopic *Pax7 *positive cells co-expressing *Pax3*. Ectopic patches of *Pax3 *expression are restricted to the right, electroporated half of the mesencephalic vesicle. (**B**') is a higher magnification of (**B**) with the GFP fluorescent image superimposed onto the preparation. (**B**") shows the distribution of Pax7-GFP expressing cells in the specimen shown in (**B**'). (**C**) Quantification of the results: percent specimens with induced (green bars) and unaltered (blue bars) *Pax3 *expression following targeted electroporation of pMES-*Pax7*. (**D**) Flat mount preparation of a HH18 chick neural tube, 24 hours after electroporation of 2 μg/μl pMES-*Pax7*. (**E**) Targeted misexpression of pMES-*Pax7 *into the ventral mesencephalic vesicle showing cells ectopically expressing *Pax3 *within the *Nkx6.1 *domain. (**E**') is a higher magnification of (**E**). fb: forebrain; fp: floor plate; mes: mesencephalic vesicle; met: metencephalic vesicle; ov: optic vesicle; otv: otic vesicle. Scale bar (**B, B**'): 100 μm.

To investigate a possible relationship between *Pax3 *and *Pax7 *in the midbrain, we ectopically delivered an expression plasmid carrying *Pax7 *together with *GFP *(pMES-*Pax7*) by in ovo electroporation into the right lateral wall of the mesencephalic vesicle at HH9-HH11 (7-13 somite stage, Figure 2A). Due to the shot-gun nature of this gene delivery method, random patches of GFP expression, indicative of groups of cells forced to express the GFP and Pax7 transgenes, were seen across the right mesencephalic wall (Figure 2A, B"). Consistent with a previous report, we found that in these patches *Pax3 *expression was strongly induced as early as 16 hours following *Pax7 *misexpression (HH14, *n *= 15/19 GFP expressing specimens exhibited strong ectopic *Pax3 *expression following electroporation of 2 μg/μl pMES-*Pax7 *(79%); Figure 2B, B', C) [11]. Robust upregulation of *Pax3 *expression was still visible 24 hours and 36 hours after pMES-*Pax7 *transfection (HH18 and HH21 respectively, Figure 2D and data not shown). Ectopic *Pax3 *expression was also observed when *Pax7 *misexpression was specifically targeted to the ventral midbrain (*n *= 8/14 GFP positive specimens exhibited ectopic *Pax3 *expression after targeted *Pax7 *expression into the ventral mesencephalic vesicle (57%); Figure 2E). Scattered groups of cells expressing elevated levels of *Pax3 *transcripts were not only located within the territory of the endogenous *Pax3 *domains, but also reached ventrally towards the *Nkx6.1 *expression domain (Figure 2B', E'). A comparison of Figure 2B' and Figure 2B" revealed that apparently not all *Pax7*-GFP positive cells also express *Pax3 *mRNA, especially not in the ventral midbrain. This may be due to the short incubation time of the embryos after electroporation (HH9-11 to HH14) and may thus reflect incomplete upregulation of *Pax3 *by *Pax7*. In addition, gene delivery by in ovo electroporation causes targeted cells to take up varying amounts of DNA. Considering that ectopic induction of *Pax3 *may need a certain threshold of *Pax7 *protein (especially in the ventral neural tube, where *Sonic hedgehog *signaling promotes the induction of ventral cell fates [25,26]), it is possible that the ectopic *Pax7 *levels may not have reached the threshold necessary for *Pax3 *induction in all GFP-positive cells. In any case however, ectopic *Pax3 *expressing cells in the ventral mesencephalon were consistently positive for *Pax7*.

**Figure 3:**
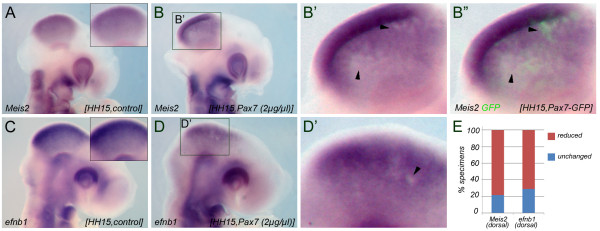
***Pax7 *represses *Meis2 *and *efnb1 *in the mesencephalic vesicle**. (**A-B**") *Meis2 *expression in HH15 chick embryos electroporated with 2 μg/μl of a control vector carrying only GFP (**A**) or 2 μg/μl pMES-*Pax7 *(**B, B**") into the right wall of the mesencephalic vesicle. The insert in (**A**) shows a higher magnification and continuous expression of *Meis2 *in the control embryo. (**B**') is a higher magnification of the boxed area in (B). Arrowheads point to patches of cells with reduced *Meis2 *transcripts. In (**B"**) the GFP fluorescence of the specimen shown in (**B **and **B**') is superimposed to visualize the extent of Pax7/GFP transfection. (**C-D**') *efnb1 *expression in a HH15 chick embryo transfected with a control vector (**C**) or with pMES-*Pax7 *(**D, D**') under identical experimental conditions. (**D**') is a higher magnification of (**D**). (**E**) Quantification of the results: percent specimens with reduced (red bars) and unaltered (blue bars) *Meis2 *or *efnb1 *expression following targeted electroporation of pMES-*Pax7*.

Two lines of evidence from our previous study suggested that *Meis2 *may not act upstream, but rather downstream of or in parallel to *Pax3 *and *Pax7*: (i) electroporation of a function blocking form of *Meis2 *into the mesencephalic vesicle had not affected *Pax3 *or *Pax7 *expression and (ii) ectopic delivery of *Meis2 *into the diencephalon was not sufficient to induce expression of either Pax gene (Figure S4 of [17]). To explore a reciprocal relationship of the three proteins, we assessed *Meis2 *expression following *Pax7 *misexpression. Contrary to our initial expectation, *Meis2 *specific transcripts were reduced in the midbrain vesicle after forced expression of *Pax7 *compared to embryos transfected with GFP alone, (*n *= 7/9 embryos exhibited reduced *Meis2 *expression following electroporation of 2 μg/μl pMES-*Pax7 *(78%); Figure 3A, B-B", E). Likewise, expression of *ephrinB1 *(*efnb1*), a downstream target of *Meis2 *in the tectal anlage, was also diminished in *Pax7 *electroporated embryos (*n *= 5/7 (71%); Figure 3C, D-D', E) [17]. Thus, although all three genes are co-expressed within the tectal anlage, elevating *Pax7 *expression levels increased *Pax3 *but repressed *Meis2 *expression under identical experimental conditions.

### Midbrain *Meis2- *and *Pax7 *expression are repressed by *Pax3 *in a dose dependent manner

**Figure 4:**
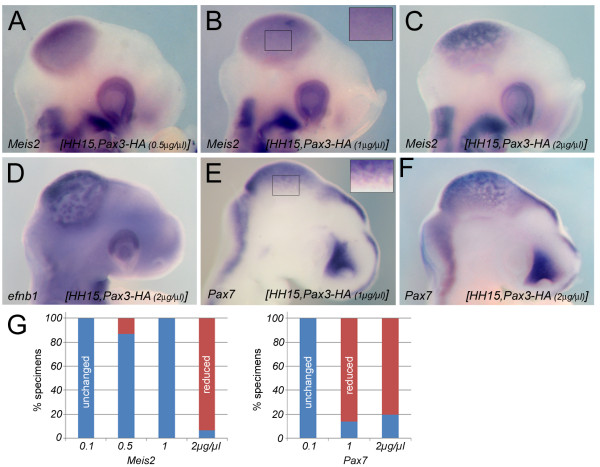
**Dose-dependent repression of *Meis2, efnb1 *and *Pax7 *by *Pax3***. (**A-C**) *Meis2 *expression in HH15 embryos electroporated with indicated concentrations of pMIWIII-*Pax3*. Loss of *Meis2 *transcripts can be seen in random patches of the electroporated right half of the mesencephalic vesicle only after transfection of 2 μg/μl of pMIWIII-*Pax3 *(**C**). (**D**) Loss of *efnb1 *transcripts following transfection of 2 μg/μl pMIWIII-*Pax3*. (E, F) *Pax7 *expression in the mesencephalic vesicle upon transfection of 1 μg/μl (**E**) and 2 μg/μl (**F**) pMIWIII-*Pax3*. The boxed areas in (**B, E**) are shown at a higher magnification in the upper right corners of the respective panels. (**G**) Quantification of the results: percent specimens with reduced (red bars) and unchanged (blue bars) expression of *Meis2 *(left) or *Pax7 *(right) following electroporation of different concentrations of pMIWIII-*Pax3*.

To further explore this apparent contradiction, we ectopically expressed a HA-tagged form or *Pax3 *(pMIWIII-*Pax3-HA*) in the mesencephalic vesicle. *Meis2 *expression was generally not affected following electroporation of concentrations between 0.1 to 1 μg/μl of the *Pax3 *expressing plasmid (*n *= 0/7 embryos exhibited reduced *Meis2 *expression following electroporation of 0.1 μg/μl pMIWIII-*Pax3-HA *(0%); *n *= 1/8 following electroporation of 0.5 μg/μl (13%); *n *= 0/8 following electroporation of 1 μg/μl (0%); Figure 4A, B, G). In contrast, transfection of 2 μg/μl of pMIWIII-*Pax3-HA *effectively repressed *Meis2 *transcripts (*n *= 13/14 (93%); Figure 4C, G). Electroporation of 2 μg/μl of pMIWIII-*Pax3-HA *also repressed *efnb1 *expression (*n *= 7/9 (78%), Figure 4D). Notably, *Pax7 *specific transcripts were lost in the dorsal midbrain already at a concentration of 1 μg/μl pMIWIII-*Pax3-HA *(1 μg/μl: *n *= 6/7 (86%); 2 μg/μl: *n *= 8/10 (80%); Figure 4E, F, G and data not shown). Thus, *Pax7*, like *Meis2*, is negatively regulated by *Pax3*, but repression of *Pax7 *occurs already at half the concentration of the *Pax3 *expressing plasmid required to repress *Meis2 *or its downstream gene *efnb1*. In summary, these results suggest that *Pax7 *induces *Pax3 *and *Pax3 *represses *Pax7 *in the dorsal midbrain leading to tightly balanced expression levels of both proteins, which in turn are permissive for expression of *Meis2 *and its target gene *efnb1*.

### Meis2-Pax3 and Meis2-Pax7 containing protein complexes exist in the tectal anlage

**Figure 5:**
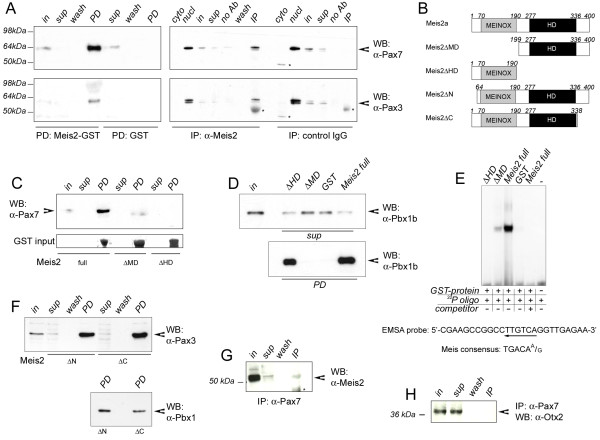
**Meis2 containing protein complexes in the mesencephalic vesicle**. (**A**) Co-precipitation of Pax3 and Pax7 with Meis2 in GST-pull down (left panels) and immunoprecipitation experiments using a Meis2 specific antibody (right panels). Upper panel: Western Blot with an antibody directed against Pax7; lower panel: Western Blot against Pax3. (**B**) Schematic representation of the Meis2 deletions N-terminally fused to GST that were used in (**A, C, D, E**, and **F**). (**C**) Pull down experiments using full length Meis2 (Meis2_[1-400]_), Meis2 lacking the MEINOX-domain (Meis2_[199-400]_; ΔMD) or Meis2 lacking the homeodomain (Meis2_[1-190]_; ΔHD) probed for Pax7. (**D**) GST-pull down with full length Meis2, ΔMD, ΔHD and GST probed for Pbx1b. (**E**) Specific DNA complex formation on a 26-bp oligonucleodite and purified GST proteins. Complex formation is competed by specific oligomers at a 10×-molar ratio ('competitor'). The sequence of the ^23^P-labeled oligomer is indicated below. (**F**) GST-pull down with ΔN and ΔC probed for Pax3 (upper panel) and Pbx1b (lower panel). (**G**) Immunoprecipitation using a Pax7-specific antibody probed for Meis2. (**H**) The same blot as in (**G**) stripped and re-probed for Otx2. Cyto: cytoplasmic extract; In: input; IP: immunoprecipitate; no Ab: precipitation with protein G sepharose beads omitting the antibody; nucl: nuclear extract; PD: pull down; sup: supernatant; wash: final wash step of the precipitates; WB: Western blot: The asterisks in (**A**) and (**C**) mark unspecific bands

As we reported previously, Meis2 forms heteromeric complexes with Otx2 in the tectal anlage and binding to Meis2 is necessary for Otx2 to induce ectopic tectal development upon misexpression in the metencephalic alar plate [17]. Based on these observations and because of the two proteins only *Meis2 *expression is specific for the prospective optic tectum (*Otx2 *is present in the entire neural tube anterior of the MHB), we had suggested that complex formation with Meis2 may confer tectal specificity to Otx2 in the dorsal midbrain, where both transcription factors are co-expressed. *Pax3 *and *Pax7 *can also trigger ectopic tectal development when misexpressed [11]. However, like for *Otx2*, their expression is not specific for the tectal anlage, but includes the alar plate along most of its length [11]. We therefore decided to examine whether Meis2 may interact with Pax3 or Pax7 in the dorsal mesencephalic vesicle. In GST-pull down experiments using a GST-tagged form of Meis2 and protein extracts prepared from HH 15-18 chick mesencephalic vesicle Pax3 and Pax7 readily precipitated with Meis2-GST but not with GST alone. Pax3 and Pax7 can thus associate with recombinant Meis2 (Figure 5A, left half, top and bottom panels). When Meis2-specific antibodies were used to precipitate endogenous Meis2-containing protein complexes Pax3 and Pax7 were successfully enriched in the precipitates. Isotype-specific control antibodies, however, were not successful (Figure 5A, right half, top and bottom panels). Precipitation of Pax7 was significantly reduced when a truncated form of Meis2 lacking the MEINOX domain fused to GST (Meis2_[199-400]_, ΔMD) was used and completely abolished when a truncated form lacking the homeodomain fused to GST (Meis2_[1-190]_, ΔHD; Figure 5B, C) was used.

To confirm that both truncated forms are active, we tested them for their ability to bind to the PBC class homeodomain protein Pbx1, a known Meis interacting partner, or to DNA respectively. Meis proteins bind to PBC class proteins via their MEINOX domain, a bipartite protein interaction domain located N-terminal of the homeodomain (Figure 5B) [27]. Accordingly, full length Meis2 and Meis2ΔHD successfully precipitated Pbx1b, whereas Meis2ΔMD and GST were ineffective (Figure 5D). Meis family proteins recognize variations of the consensus sequence motif 5'-TGATA(A/G)-3' in the regulatory regions of target genes [28]. Although Meis proteins frequently cooperate with PBC class proteins in binding to DNA, examples of Pbx independent DNA binding are also known [29]. We therefore performed electromobility shift assays (EMSA) with purified Meis2-GST fusion proteins and a ^32^P-labeled oligonucleotide probe, which was previously shown to be bound by Meis independently of Pbx [29]. Complex formation of the probe was observed with full-length Meis2 and Meis2ΔMD but not with Meis2ΔHD or GST alone (Figure 5E). Excess of a non-labeled specific oligonucleotide effectively competed for full length Meis2 or Meis2ΔMD DNA binding (Figure 5E and data not shown).

GST-fusion proteins lacking the amino acids N-terminal to the MEINOX domain (Meis2_[64-400]_ΔN) or Meis2 lacking the transactivation domain, which is located C-terminal to the homeodomain (Meis2_[1-338]_ΔC), also effectively precipitated Pax3 and Pbx1b (Figure 5F) [17,30]. We therefore conclude that the MEINOX domain and homeodomain of Meis2 are involved in the association with Pax3 and Pax7. Complex formation between Meis2 and Pax7 was also seen when an antibody specific for Pax7 was used in the immunoprecipitation experiments (Figure 5G). The relatively weak Meis2-specific band in the IP with the Pax7-specific antibody compared to the robust co-precipitation of Pax7 with the Meis2-specific antibody may indicate that only a fraction of the Meis2 protein present in the extracts is bound to Pax7 (compare immunoprecipitate with α-Meis2 in Figure 5A (upper right panel) and immunoprecipitate with Δ-Pax7 in Figure 5G). To test whether Meis2 forms multimeric complexes with Pax7 and Otx2, we re-probed the blot with an antibody directed against Otx2 (Figure 5H). Contrary to Meis2, Otx2 was not detected in the immunoprecipitate. We were also not able to precipitate Pax7 with an Otx2 specific antibody (data not shown). Although we cannot rule out that the antibodies used in our precipitation experiments may, to different extents, interfere with the ability of their antigens to engage in multiprotein interactions, our results argue against the existence of large multimeric complexes involving Meis2, Otx2 and Pax7. Instead, we propose that Meis2 may form heteromeric complexes with either Pax3 or Pax7 or Otx2 in the dorsal mesencephalic vesicle.

## Discussion

Based on in ovo electroporation and immunoprecipitation experiments in chick embryos, we here provide experimental evidence for reciprocal regulation and subsequent physical interaction of *Pax3, Pax7 *and *Meis2 *in the tectal anlage.

### Dose-dependent and reciprocal regulation of Pax3 and Pax7 in the tectal anlage

Interdependent regulation between *Pax3 *and *Pax7 *has been reported in other physiological contexts before. *Pax7 *transcripts, for instance, are upregulated in the embryonic dorsal spinal cord of *Splotch *mice, a naturally occurring mutant of *Pax3*, suggesting that in mouse spinal cord *Pax3 *normally functions to repress *Pax7 *[14]. In *Xenopus laevis *embryos, on the other hand, expression of *Pax7 *and *Pax3 *in the neural tube and neural crest strikingly differs from that in mice and chicks, which indicates different functional specifications of both genes in amphibians compared to mammals and birds [12]. Indeed, Morpholino-mediated knock down of *Pax3 *or misexpression of a function blocking form of *Pax3 *reduced *Pax7 *transcript levels in the *Xenopus *spinal cord, suggesting that *Pax3 *positively regulates *Pax7 *in amphibians [12]. Although species specific differences obviously exist, in summation these results argue for interdependent regulation of *Pax3 *and *Pax7 *in the spinal cord. The observations reported in the present study extend this reciprocal regulation of both Pax proteins to the embryonic mesencephalon and point to a possible dose-dependent function of *Pax3 *during tectal development. Since *Pax3 *and *Pax7 *are closely related and forced expression of either molecule in the diencephalon leads to identical cell fate changes, *Pax3 *and *Pax7 *were suggested to play redundant functions during tectal development [11,31]. The cross-regulation between *Pax3 *and *Pax7 *we report here may allow for a tight, mutual regulation of both gene products, which prevents over-activation of shared downstream pathways and at the same time permits functional compensation if one of the genes is mutated.

Examples for dose dependent functions of transcriptional regulators are well known in invertebrate development. In the early *D. melanogaster *embryo, for instance, spatial gradients of transcription factors control the expression of distinct sets of target genes, which ultimately control morphogenesis [32-34]. Similar dose dependent activities have also been reported for transcription factors that engage in reciprocal regulation. Oligodendrocyte differentiation in the embryonic vertebrate spinal cord, for instance, depends on the concerted activities of *Olig2, Sox11 *and *Nkx2.2*. This process not only requires mutual regulation of the three proteins but also depends on the gene dosage of *Olig2*, as both haploinsufficiency and overexpression of *Olig2 *significantly delayed oligodendrocyte maturation [35]. Dosage dependent developmental defects also exist for the paired-type transcription factor *Pax6 *in both human and mice [36,37]. Mice heterozygote for PAX6 (*Sey, small eye*) display ocular defects with small eyes and malformations of the anterior eye chamber, as do mice carrying multiple copies of a PAX6-containing YAC (yeast artificial chromosome) [38-40]. The underlying mechanism of such dosage dependent requirement of transcriptional regulators is only partially understood at present. One evident explanation takes into account that transcription factors frequently function in the context of larger multiprotein complexes, which involve other DNA binding proteins as well as transcriptional co-regulators that modulate chromatin dynamics. Since the stoichiometry of these transcriptional complexes must be tightly controlled, too much or too little of any given component may disturb the formation of functional complexes and consequently result in insufficient transcriptional activity. Changing the intracellular concentration of individual transcription factors into either direction may therefore adversely affect expression of their target genes and ultimately lead to similar developmental defects.

**Figure 6:**
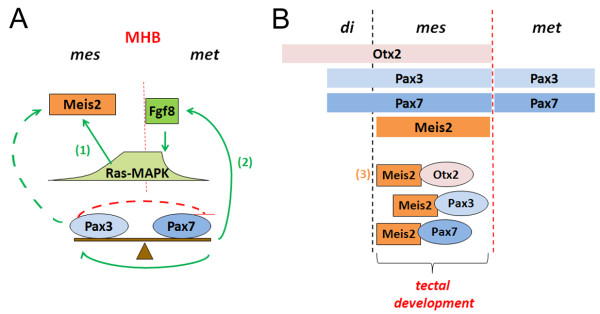
**Model for a possible cooperation of *Meis2, Pax3, Pax7 *and *Otx2*during tectal development**. See text for details. Red lines indicate negative regulation, green arrows positive regulation. Dashed lines indicate hypothetical direct regulation of the Meis2 promoter/enhancer by different Pax3 concentrations. Solid lines indicate indirect regulation of Meis2 expression via Pax3/7 mediated induction of Fgf8 as previously reported: (1) Regulation of Meis2 expression in response to Ras-MAPK signaling levels reported in [19]; (2) Induction of Fgf8 by Pax3 and Pax7 reported in [11]; (3) Existence of Meis2-Otx2 containing protein complexes in the tectal anlage reported in [17].

An intriguing second explanation of how the net activation of an enhancer can be directly linked to different concentrations of a given transcription factor comes from studies of transcription factor gradients in early *D. melanogaster *embryos. Here, the *bicoid *protein binds individual recognition sites in the regulatory region of its target gene *hunchback *with different affinities. This ensures that high-affinity binding sites can already be bound and activated by low concentrations of the protein, whereas high *bicoid *levels are needed to activate low-affinity sites [41]. Likewise, Rowan and colleagues recently reported that the PAX6 lens enhancer was synergistically regulated by multiple Prep1 proteins, each non-cooperatively bound to a low-affinity binding site [42]. Both examples provide a mechanism of how different concentrations of a given transcription factor can be directly translated into the net activity of a target enhancer. *Pax3 *may therefore directly modulate *Pax7 *and *Meis2 *expression, potentially by binding to target sides in the enhancers of each gene that differ in their relative affinity for *Pax3 *(Figure 6A, dashed lines). A detailed comparison of the midbrain-specific regulatory regions of *Meis2 *and *Pax7 *would be needed to test this hypothesis. However, although upstream regulatory elements of *Pax7 *have been identified, none of them faithfully recapitulates expression in the mesencephalic alar plate and *Meis2 *enhancer elements have remained elusive so far [43]. Nevertheless it is worth pointing out that recognition sequences with different binding affinities for *Pax3 *have been identified in vitro and that differences in the affinity of *Pax3 *to these sites mediate varying levels of transactivation [44].

Alternatively, *Pax3 *and *Pax7 *may control *Meis2 *expression indirectly (Figure 6A, solid lines). This model takes into account that both Pax proteins were shown to trigger expression of MHB associated genes, including *Fgf8*, when ectopically expressed in the anterior neural tube [11]. We have previously found that *Meis2 *transcriptional activation requires low levels of Ras-MAPK pathway activity that are characteristic for the mesencephalic vesicle, but is inhibited by strong Ras-MAPK signals, which induce metencephalic fate specification [19]. Hence, *Pax3 *and *Pax7 *might impinge on *Meis2 *expression indirectly through modulating *Fgf8 *expression levels (and consequently the resulting strength of Ras-MAPK pathway activation) at the MHB organizer. Irrespective of whether *Pax3 *and *Pax7 *act directly or indirectly on *Meis2 *expression, interdependent and balanced expression of both paired proteins may serve to prevent excessive autoactivation of *Meis2 *in the tectal anlage.

### Multiple transcriptional regulators associate with Meis2 in the tectal anlage

Functional subdivision of the brain is preceded by the restricted expression of transcriptional regulators at neural tube stages. The anlagen of the optic tecta, originating from the alar plates of the mesencephalic vesicle, for instance are characterized by the combinatorial expression of *Otx2, Pax3, Pax7*, and *Meis2 *(Figure 6B). Notably, all four proteins can instruct tectal fate when ectopically delivered to the alar plate of adjacent brain vesicles, but only *Meis2 *expression faithfully demarcates the prospective optic tecta. *Otx2 *expression encompasses the entire neural tube anterior of the MHB and *Pax3 *and *Pax7 *are present in the alar plates along most of the neural tubes anterior-posterior axis [11,17,45].

As we demonstrated previously, Meis2 forms heteromeric complexes with Otx2 in the mesencephalon and association with *Meis2 *can restore full transcriptional activity of *Otx2 *in the presence of the co-repressor Tle4 in an *Otx2 *dependent reporter assay [17]. This observation prompted us to suggest that *Meis2 *may act as tectum-specific cofactor of *Otx2*. We can now extend this observation to include *Pax3 *and *Pax7*. Association of Meis2 with Pax3 and Pax7 was observed in vitro and endogenous Meis2-Pax containing protein complexes could be precipitated from tectal tissue. Notably however, we failed to detect interaction of Pax7 and Otx2, suggesting that Meis2 forms individual complexes with each of these proteins.

The identification of Meis2-Pax containing nuclear complexes has also implications for the general concept of TALE-HD protein function. All Pax proteins except Pax4 and Pax6 contain an octapeptide motif, a conserved stretch of eight amino acids related to the eh1 domain. The eh1 domain mediates transcriptional repressor activity through recruitment of co-repressors of the Tle/Grg family. Indeed, Pax3 can directly associate with Grg4 or other transcriptional co-repressors and has been implicated in transcriptional repression in several physiological contexts [46-48]. The Meis2 - Pax3/7 interaction reported here suggests that Meis2 can be part of transcriptional activator as well as repressor complexes. In this context it is worth noting that tectal fate specification not only requires the activation of tectum specific genes, but also the repression of competing cellular fates. Indeed, *Meis2 *is not only positively regulates the expression of tectum associated genes such as *efnb1 *or *Dbx1*, but also represses the diencephalic marker gene *Pax6 *[17].

## Conclusion

We have previously shown that the TALE-homeodomain protein *Meis2 *acts downstream of the MHB organizer and controls tectal development by cooperating with *Otx2*. The results described here expand this view and suggest that tectal *Meis2 *expression levels are modulated by *Pax3 *and *Pax7 *and that the expression levels of both Pax proteins have to be tightly balanced to allow for expression of *Meis2*. In addition, we find that Meis2 not only associates with Otx2 in dorsal mesencephalic vesicle but also with Pax3 and Pax7. Meis2 is the only known transcriptional regulator so far that is able to instruct tectal fate specification and whose expression specifically marks the tectal anlage at mid to late somite stages. We therefore propose that spatially controlled association with Meis2 may serve as a general mechanism to confer tectal specificity to a wide range of otherwise broadly expressed transcription factors.

## Methods

### Expression constructs, in ovo electroporation

Full length coding regions of *Pax3 *and *Pax7 *were cloned by RT-PCR from total RNA of HH16-20 chick optic tecta (primer sequences available upon request) and corresponded to NCBI Acc# NM_204269 (*Pax3*) and NCBI Acc# NM_205065 (*Pax7*) respectively. To generate pMIWIII-*Pax3-HA*, the coding region of chick *Pax3 *was fused to a triple HA tag and cloned into the chick expression vector pMIWIII [49]. Full length *Pax7 *was subcloned into pMES, which contains an IRES-eGFP (internal ribosome entry site - enhanced green fluorescent protein) cassette to allow Pax7 expression together with GFP, resulting in pMES-*Pax7 *[50]. Unless otherwise noted, 2 μg/μl of each construct were electroporated into the right wall of the neural tube of HH 9-11 chick embryos as described (White Leghorn) [22,51]. In the case of pMIWIII 0.5 μg/μl pMIWIII-*GFP*, expressing enhanced green fluorescent protein, was co-electroporated for visualization. pMIWIII-*GFP *or pMES served as controls. All experiments involving fertilized chick eggs were performed in accordance with the guidelines of the local animal care committee.

### In-situ hybridization

In-situ hybridization on vibratome sections or whole embryos was performed as described in [22,51]. The cDNAs used to generate *in-situ *probes for *Meis2, efnb1, Pax3, Pax7*, and *Nkx6.1 *were gifts from D. O'Leary (Salk Institute, La Jolla, CA, USA), H. Rohrer (Max Planck Institute for Brain Research, Frankfurt, Germany), P. Gruss (Max Planck Institute for Biophysical Chemistry, Göttingen, Germany), J. Rubenstein (UCSF, San Francisco, CA, USA) or were cloned from chick HH10-12 whole head total RNA by RT-PCR with gene specific primers (primer sequences are available upon request).

### Isolation and analysis of Meis2 interacting proteins

#### Preparation of tectal lysates

Approximately 30 HH15-18 chick tecta per experiment were lysed in 10 mM Hepes pH8, 10 mM KCl, 0.1 mM EDTA, 2 mM DTT, 1% Igepal (Sigma Aldrich, Germany), and Complete™ protease inhibitor tablets (Roche, Germany). Cell nuclei were collected by brief centrifugation. The supernatant contained the cytosolic fraction ('*cyto*'). The cell nuclei were reconstituted in 10 mM Hepes pH8, 10 mM KCl, 0.1 mM EDTA, 2 mM DTT, 400 mM NaCl, 1% Igepal and Complete™ protease inhibitors and incubated for 15 min at 4°C under constant rotation. Cellular debris was removed by brief centrifugation ('*nucl*'). Cytosolic and nuclear fractions were combined (designated input, '*in*'). Lysates were pre-cleared by incubation with empty glutathione sepharose 4B beads (GE Healthcare-Amerham, NJ) or empty Protein-G agarose beads (Roche, Germany) for 30 min to 1 hour under constant rotation at 4°C.

#### GST-pull down experiments

Full length *Meis2a *(*Meis2a*_[1-400]_), the truncated variants *Meis2a*_[1-190] _(lacking the C-terminus including the homeodomain, ΔHD), *Meis2a*_[199-400] _(lacking the N-terminus including the MEINOX-domain, ΔMD), *Meis2a*_[64-400] _(lacking the N-terminus but retaining the MEINOX domain, ΔN) or *Meis2a*_[1-338] _(lacking the C-terminal transcriptional activation domain but retaining the homeodomain, ΔC) were N-terminally fused to glutathione S-transferase (GST). GST-fusion proteins were purified following standard procedures. Immobilized GST-fusion proteins were incubated with pre-cleared tectal lysates for 2 hours under constant rotation at 4°C. Following extensive washes in 10 mM Hepes pH8, 10 mM KCl, 0.1 mM EDTA, 2 mM DTT, 150 mM NaCl, 1% Igepal and Complete™, the protein complexes were analyzed by SDS-PAGE followed by Western Blot following standard procedures.

#### Co-immunoprecipitation assay

Pre-cleared tectal lysates were incubated with polyclonal anti-Meis2 antibody (generously provided by Dr. Arthur Buchberg, Thomas Jefferson University Medical School, Philadelphia) or monoclonal anti-Pax7 antibody (Developmental Study Hybridoma Bank, Iowa City, IA) overnight at 4°C under constant rotation. Protein-G agarose beads (Roche, Germany) were added for 1 hour at 4°C rotating. After extensive washes, the immunoprecipitates were separated by SDS-PAGE and analyzed by Western Blot. For Western Blot monoclonal anti-Pax3 (mouse, DHSB, IA; 1:5); monoclonal anti-Pax7 (mouse, DHSB, IA; 1:5); polyclonal Otx2 (goat, R&D Systems, MN; 1:2000), or polyclonal anti-Meis2 (rabbit, A. Buchberg, 1:30.000) were used.

### Electromobility shift assays

Radioactively labeled oligonucleotide probes (5'-CGAAGCCGGCCTTGTCAGGTTGAGAA-3') were generated by annealing complementary single-strand oligonucleotides in a solution containing 10 mM Tris pH7.5 and 20 mM NaCl and labeled with polynucleotide kinase (Roche, Germany) in the presence of γ^32^P-ATP. Binding reactions typically contained 2 μg purified GST protein in a buffer containing 10 mM Tris HCl (pH 8.0), 150 mM KCl, 0.25 mM EDTA, 12,5% glycerol, 0.2 mM DTT, Complete™ protease inhibitor cocktail, 1 μg/μl bovine serum albumin and 1 μg poly(dIdC) and were incubated for 10 minutes at room temperature. 20.000 cpm of the labeled probe were added and the reaction was incubated for further 30 minutes. For competition experiments, a 10-fold molar excess of the non-labeled oligomer was mixed with the radiolabeled probe prior to addition of the proteins. DNA and DNA-protein complexes were resolved on 6% non-denaturing polyacrylamide gels.

## Authors' contributions

ZA carried out the in ovo electroporation and in situ hybridization experiments (except Figure 2) as well as the biochemical analyses. NL performed the experiments shown in Figure 2. AH contributed to the electroporation experiments. DS and AW designed and coordinated the study, DS wrote the manuscript. All authors read and approved the final manuscript.
